# XPC and POLH/XPV Genes Mutated in a Genetic Cluster of Xeroderma Pigmentosum Patients in Northeast Brazil

**DOI:** 10.3389/fgene.2021.784963

**Published:** 2022-01-17

**Authors:** Ligia Pereira Castro, Danilo Batista-Vieira, Tiago Antonio de Souza, Ana Rafaela de Souza Timoteo, Jessica Dayanna Landivar Coutinho, Isabel Cristina Pinheiro de Almeida, Sheila Ramos de Miranda Henriques, Fabio Medeiros de Azevedo, Reginaldo Cruz Alves Rosa, Patricia L Kannouche, Alain Sarasin, Carlos Frederico Martins Menck, Tirzah Braz Petta

**Affiliations:** ^1^ DNA Repair Laboratory, Department of Microbiology, Institute of Biomedical Sciences, University of São Paulo, São Paulo, Brazil; ^2^ Department of Cell Biology and Genetics Federal University of Rio Grande do Norte, Natal, Brazil; ^3^ Instituto de Ensino, Pesquisa e Inovação, Liga Contra o Câncer, CECAN, Natal, Brazil; ^4^ Department of Genetics, Ribeirão Preto Medical School, University of São Paulo, Ribeirão Preto, Brazil; ^5^ UMR9019 - CNRS, Genome Integrity and Cancers, Université Paris-Saclay, Gustave Roussy, France; ^6^ Department of Pathology, Hoffman Medical Research Building, USC Keck School of Medicine, Los Angeles, CA, United States; ^7^ Clinical Pathology and Cytology, Karolinska Institute Radiumhemmet, Karolinska University Hospital in Solna, Stockholm, Sweden

**Keywords:** genetic cluster, xeroderma pigmentosum, molecular diagnosis, DNA repair, skin cancer

## Abstract

Xeroderma pigmentosum (XP) is a rare genetic condition in which exposure to sunlight leads to a high tumor incidence due to defective DNA repair machinery. Herein, we investigated seven patients clinically diagnosed with XP living in a small city, Montanhas (Rio Grande do Norte), in the Northeast region of Brazil. We performed high-throughput sequencing and, surprisingly, identified two different mutated genes. Six patients carry a novel homozygote mutation in the *POLH/XPV* gene, c.672_673insT (p.Leu225Serfs*33), while one patient carries a homozygote mutation in the *XPC* gene, c.2251-1G>C. This latter mutation was previously described in Southeastern Africa (Comoro Island and Mozambique), Pakistan, and in a high incidence in Brazil. The XP-C patient had the first symptoms before the first year of life with aggressive ophthalmologic tumor progression and a melanoma onset at 7 years of age. The XP-V patients presented a milder phenotype with later onset of the disorder (mean age of 16 years old), and one of the six XP-V patients developed melanoma at 72 years. The photoprotection is minimal among them, mainly for the XP-V patients. The differences in the disease severity between XP-C (more aggressive) and XP-V (milder) patients are obvious and point to the major role of photoprotection in the XPs. We estimate that the incidence of XP patients at Montanhas can be higher, but with no diagnosis, due to poor health assistance. Patients still suffer from the stigmatization of the condition, impairing diagnosis, education for sun protection, and medical care.

## Introduction

Xeroderma pigmentosum is an autosomal recessive disease in which the patients develop a high frequency of skin cancer, internal tumors, and neurological abnormalities (20–30% of the patients) (Menck and Munford, 2014). The tumor susceptibility is due to germline mutations that impair the processing of DNA lesions by nucleotide excision repair (NER) or by the translesion synthesis (TLS) pathway. The initial recognition step distinguishes two subpathways of NER. The transcription-coupled repair (TCR), in which the recognition occurs on the transcribed strand by the arrest of the RNA polymerase, and the nontranscribed genome is repaired by the global genomic repair (GGR) pathway (Hanawalt, 2002). These pathways are responsible for correctly cleaning up the DNA from lesions induced mainly by the ultraviolet radiation (UVR) from sunlight. Deficiency in this process leads to genetic instability, mutagenesis, and tumor progression (Kraemer et al., 1994).

XP has a wide range of symptom severity and clinical features among the eight complementation groups (XP-A to XP-G and XP-V). Patients mutated at one of the seven genes from *XPA* to *XPG* are NER-deficient, while mutations at *POLH/XPV* lead to a deficiency in TLS, a pathway involved with damage tolerance (Menck and Munford, 2014). XP-C cells lack an essential step of the DNA damage recognition at the GGR, and the XP variant (XP-V) type is NER proficient, but lacking the DNA polymerase eta function of bypassing the UV-induced lesions. The difference in which the mechanism is not working is directly linked to the phenotype of the patient. Therefore, molecular diagnosis is critical for the management of their prognosis (Fassihi et al., 2016; Lehmann and Fassihi, 2020). XP-C, XP-E, and XP-V are mainly affected by skin and eye tumors with no neurological abnormalities. XP-C patients are seriously affected with aggressive skin tumors and more than 1,000-fold increased risk of hematological malignancies, while XP-E and XP-V patients present a milder phenotype (Fassihi et al., 2016; Sarasin et al., 2019b; Oetjen et al., 2020).

This work identified a total of 13 patients (9 alive and 4 deceased) with a clinical diagnosis of XP in the city of Montanhas (state of Rio Grande do Norte-RN-, Brazil), leading to an estimated XP frequency of 1/1,120 inhabitants, considering the total population as 11,200 inhabitants in that city (Brazilian Institute of Geography and Statistics). Three patients did not participate in the study, and probably the XP incidence is underestimated due to a stigmatization effect. The denial of the disease impairs diagnosis, medical care, genetic counseling, and prevention practices reducing their quality and life expectancy. By performing the next-generation sequencing (NGS) with a specific panel of DNA repair genes, we identified homozygous mutations in two different genes: *POLH* (six patients) and *XPC* (one patient). This is the second XP genetic cluster described in Brazil (Munford et al., 2017), but the first to identify two different mutated XP genes in such a small community.

## Patients and Methods

### Patients

We collected saliva samples from 16 individuals (14 families), 7 XP patients (mean: 48 years of age, range: 7 to 89 years), and 9 relatives (Table 1). All patients were born in the city of Montanhas (RN) and were followed at the Hospital Universitário Onofre Lopes—HUOL/UFRN. Basal cell carcinoma (BCC) and squamous cell carcinoma (SCC) records were available from 2017 to 2021.

**TABLE 1:** Family structure information from seven patients and nine relatives.

Pedigree ID	Family ID	Genealogy ID	Individual ID	Father	Mother	G	BIR-YR	Status	Age (y)	AFF
1	1	III.3	XP03RN.br	II.9	II.10	M	1931	Dead^a^	89	2
1	1	III.10	XP04RN.br	II.9	II.10	M	1935	Live	86	2
1	1	III.12	RN09.br	II.9	II.10	F	1945	Live	76	1
1	2	IV.1	XP05RN.br	III.1	III.2	M	1972	Live	49	2
1	3	IV.3	RN10.br	III.3 (XP03RN.br)	III.4 (RN14.br)	F	1959	Live	62	1
1	4	III.4	RN14.br	II.3	II.4	F	1944	Live	77	1
1	5	IV.11	XP06RN.br	III.7	III.8	F	1974	Live	47	2
1	6	IV.6	XP08RN.br	III.5 (RN16.br)	III.6 (RN17.br)	F	1983	Dead^b^	38	2
1	7	V.7	RN11.br	IV.13	IV.14	M	2002	Live	19	1
1	8	III.13	XP15RN.br	II.11	II.12	M	1945	Live	76	2
1	9	III.5	RN16.br	II.5	II.6	M	1939	Live	82	1
1	10	III.6	RN17.br	II.7	II.8	F	1955	Live	67	1
1	11	V.2	RN18.br	IV.5	IV.4	F	2009	Live	12	1
2	12	IV.1	XP07RN.br	III.2	III.3	F	2013	Live	8	2
2	13	III.3	RN12.br	II.3	II.4	F	1997	Live	25	1
2	14	III.2	RN13.br	II.1	II.2	M	1997	Live	24	1

Note. Individuals with the same family ID represent full siblings. G, gender; M, male; F, female; Status, Live or death; BIR-YR, year of birth; AFF, affected status 1 (not affected); 2 (affected).

a Year of death: 2019.

b Year of death: 2021.

This study had previous authorization approved by the Ethics Committee of the Institute of Biomedical Science at the University of São Paulo (ICB—USP), approval number #48347515.3.00005467. All saliva samples and pictures were collected with prior written informed consent, complied with legal and ethical rules.

### Molecular Analysis

The DNA Repair Laboratory received, from October 2018 to April 2019, saliva samples from 16 individuals (7 patients and 9 relatives) collected using an Oragene DNA self‐collection kit (DNA Genotek Inc., Ottawa, Canada). We extracted the genomic DNA as recommended by the protocols of the manufacturers. DNA quantity and quality were analyzed by Nanodrop-1000 (Wilmington, DE, USA) and 1% agarose gel electrophoresis. We used a customized DNA repair panel to prepare the DNA library for NGS from samples of the patients, as previously described (Menck and Munford, 2014). The alignment and variant calling were performed at Surecall software v3.5.1.46 (Agilent^®^), using the GRCh37/hg19 version of the reference human genome. Following the assertion criteria established by the DNA repair laboratory, we validated the mutations by Sanger sequencing and classified the variants according to the American College of Medical Genetics (ACMG) guidelines, using the VarSome variants search Engine (Richards et al., 2015; Kopanos et al., 2019). The DNA repair laboratory assertion criteria and the accessions for this submission (SCV001652706 - SCV001652707) can be found on the ClinVar database (ClinVar data access: https://www.ncbi.nlm.nih.gov/clinvar/submitters/500218/). We performed the segregation analysis of the mutation at the *XPC* (3p25.1; NC_000003.11; NM_004628.4) and *POLH* (6p21.1; NC_000006.11; NM_006502.2) genes by genotyping the relatives of the patient. Variants were reported at the nucleotide and protein levels as recommended by the Human Genome Variation Society (HGVS) (den Dunnen et al., 2016). The pedigrees were drawn following the Standardized Human Pedigree Nomenclature (Bennett et al., 1995).

## Results and Discussion

**FIGURE 1:**
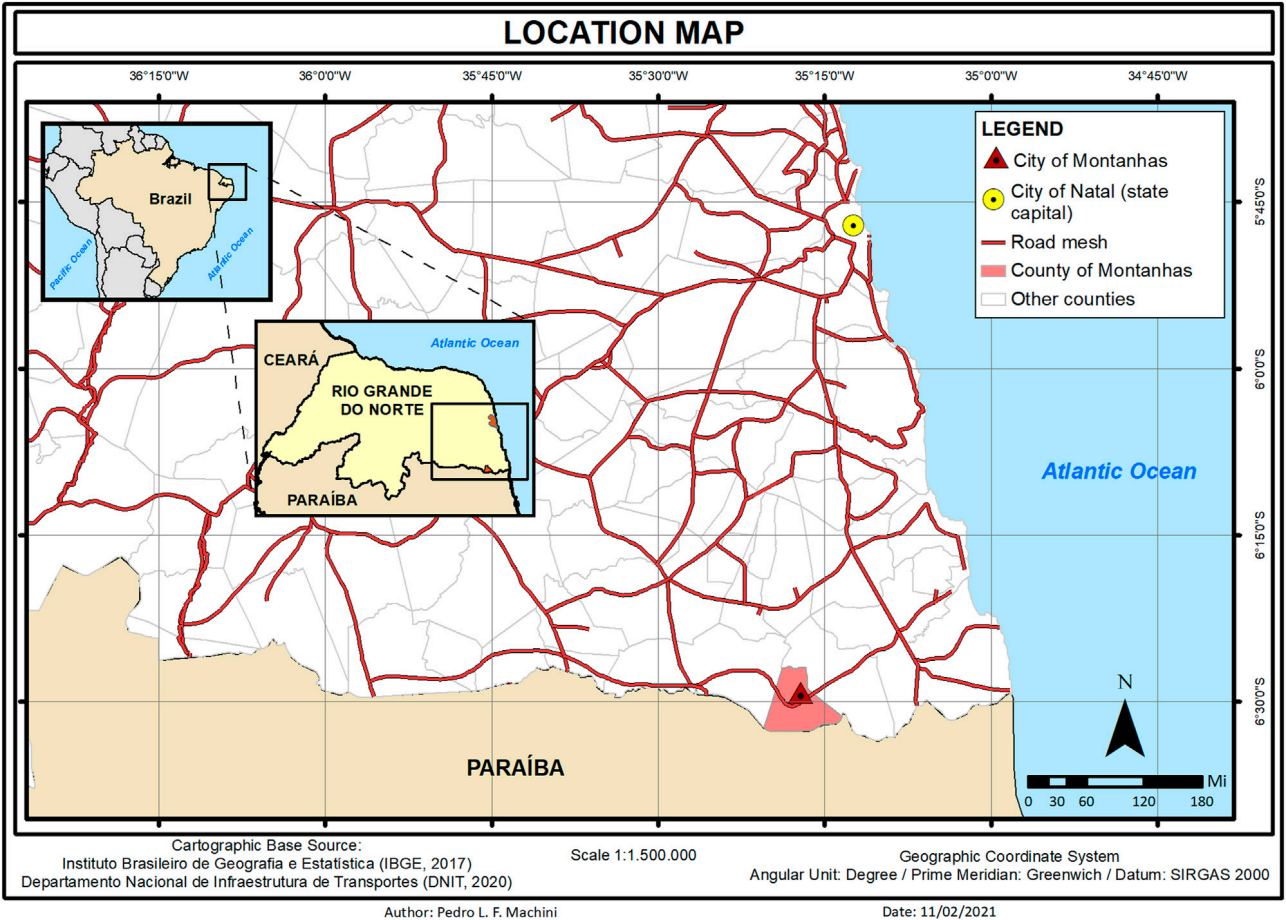
Location map from the city of Montanhas in the state of Rio Grande do Norte (RN) in the northeast of Brazil. Patients receive medical care 60 miles away in the city of Natal (capital of the state).

We provided the molecular diagnosis for 7 XP patients, out of 13, at Montanhas, a small city in the south of Rio Grande do Norte (RN), Brazil. Different from the genetic cluster previously reported at Araras (Goiás, Brazil) (Munford et al., 2017), Montanhas is not an isolated community. Natal, the state capital, is 60 miles away from Montanhas (approximately 1 h and a half of paved road by car) where patients receive medical care (Figure 1).

**FIGURE 2:**
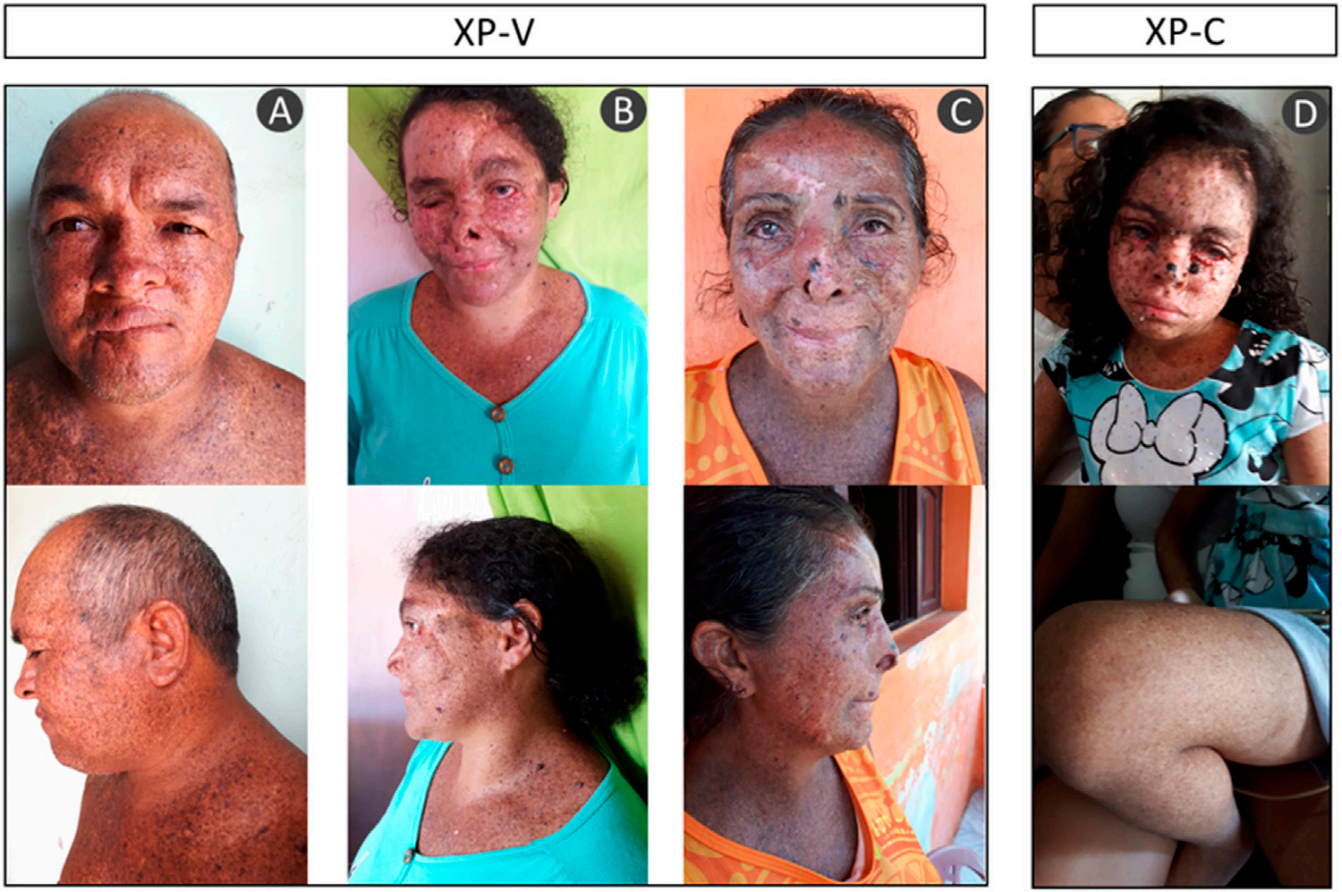
Patient phenotype from three XP-V: **(A)** XP04RN.br, **(B)** XP08RN.br, **(C)** XP06RN.br, and one XP-C **(D)** XP07RN.br. The pictures are shown with the consent of the patients.

The majority of the patients were born from consanguineous marriages. All of them presented the typical clinical phenotype of XP with no neurological abnormalities: photosensitivity, actinic keratosis, basal and squamous cell carcinomas (BCC and SCC), freckling, hyper- and hypopigmentation, and ophthalmologic abnormalities as ocular lesions and blurred vision (Figure 2 and Table 2). None of them reported sunburn after sun exposure. The different clinical symptoms comparing patient XP07RN.br and the other XPs call attention, as one would expect the same mutation affecting all patients in this small community.

**TABLE 2:** Clinical symptoms from the XP-V and XP-C patients from Montanhas (RN).

Individual ID (age)	1st Symptoms	1st Symptoms (age)	1st tumor onset (age)	No. of skin BCC	No. of skin SCC	Melanoma onset	Ocular abnormality
*XPV* NM_006502.2: c.672_673insT [p.Leu225Serfs*33]							
XP03RN.br (89 years)	Skin	20 years	20 years	5	6	0	Yes
XP04RN.br (85 years)	Skin	18 years	18 years	2	4	0	Yes
XP15RN.br (75 years)	Skin	12 years	12 years	4	5	73 years	Yes
XP05RN.br (48 years)	Skin	16 years	16 years	6	8	0	Yes
XP06RN.br (46 years)	Skin	15 years	15 years	10	3	0	Right eye enucleation (45 years)
XP08RN.br (37 years)	Skin	15 years	15 years	6	4	0	yes
*XPC* NM_004628.4: c.2251-1G>C							
XP07RN.br (7 years)	Extreme ocular photophobia	before 1 year	4	1	11	7 years	Left eye enucleation (7 years)

Note. Clinical data of BCC and SCC incidence were provided from 2017 to 2021.

Molecular genetic analysis revealed two mutations, each one in two different genes. Most of the patients (six) carry a homozygous insertion that leads to a frameshift mutation in the *POLH* gene and are, thus, XP-V (OMIM #278750). The map location of the mutation according to GRCh37/hg19 sequence reference is NC_000006.11: g.43568736_43568737insT; NM_006502.2: c.672_673insT; NP_006493.1: p.Leu225Serfs*33. The patient XP07RN.br, however, carries a homozygous mutation at a canonical splice site in the *XPC* gene (NC_000003.11: g.14190232C > G; NM_004628.4: c.2251-1G>C) and, therefore, is XP-C (OMIM #278720).

The mean age of the XP-V patients was 63 years old (range: 37–89 years), with the first symptoms (skin lesions) at 16 years (range: 12–20 years). Only one of them developed melanoma at 72 years. The oldest XP-V patient (XP03RN.br) died in 2019 at 89 years old. According to his medical record, he went to the Hospital bedridden due to a stroke. Approximately 2 weeks before, he developed edema with nodulation in the right submandibular angle, suspected of melanoma metastasis. He was treated with amoxicillin–clavulanate and died a few months later, probably not related to the XP phenotype. In October of 2021, the patient XP08RN.br died (38 years), and we did not have access to the cause of death.

The XP-C patient, XP07RN.br, presented an aggressive phenotype with the first symptoms before the first year of life with extreme photophobia and ophthalmologic sensitivity. Around 3–4 months of age, her eyes used to water a lot, with difficulties to open in bright environments. At 7 months of age, there was a medical concern about the blood result test from serum thyroid-stimulating hormone level (TSH) of 9.35 mIU/L (reference from 0 to 11 months: 0.8–6.3 mIU/L). However, the follow-up showed that the level decreased 3 months later to 4.95 mIU/L and 1 month later to 3.59 mIU/L. In 2020, at 8 years of age the level is 6.83 mIU/L, in the limit of the normal reference. Freckles got intense after 1 year old, and at 4 years, she had her first tumor onset in the left eye. As her family had to wait 1 year for the surgery due to the lack of medical assistance available in the location, the tumor grew rapidly, and unfortunately, the eye had to be enucleated.

In October 2020 (7 years), she presented a melanoma onset at the glabella region and nasal dorsum. The lymph node was not evaluated. The surgery completely removed the melanoma and she is being treated with interferon (10.000 UI/m^2^) three times a week for a total of 48 weeks. There was an excellent response to the treatment. An improvement was observed in the behavior of the child, who now is more willing to talk and interact in the clinic.

### Mutation Analysis

The *POLH* insertion c.672_673insT (p.Leu225Serfs*33) was not previously reported in an XP patient. However, a deletion at Lys224, c.672delA (p.Lys224Trpfs229), was reported in two XP patients from Algeria (Opletalova et al., 2014). The patients, XP888VI and XP968VI (siblings), who are compound heterozygote for c.672delA and c.2251-1G>C, presented a mild clinical phenotype, similar to the XP-V patients from Montanhas. Due to their genomic proximity, we used Sanger sequencing to compare these two mutations (from Brazil and Algeria) and confirmed that these are different mutations (Supplementary Figure S1).

**FIGURE 3:**
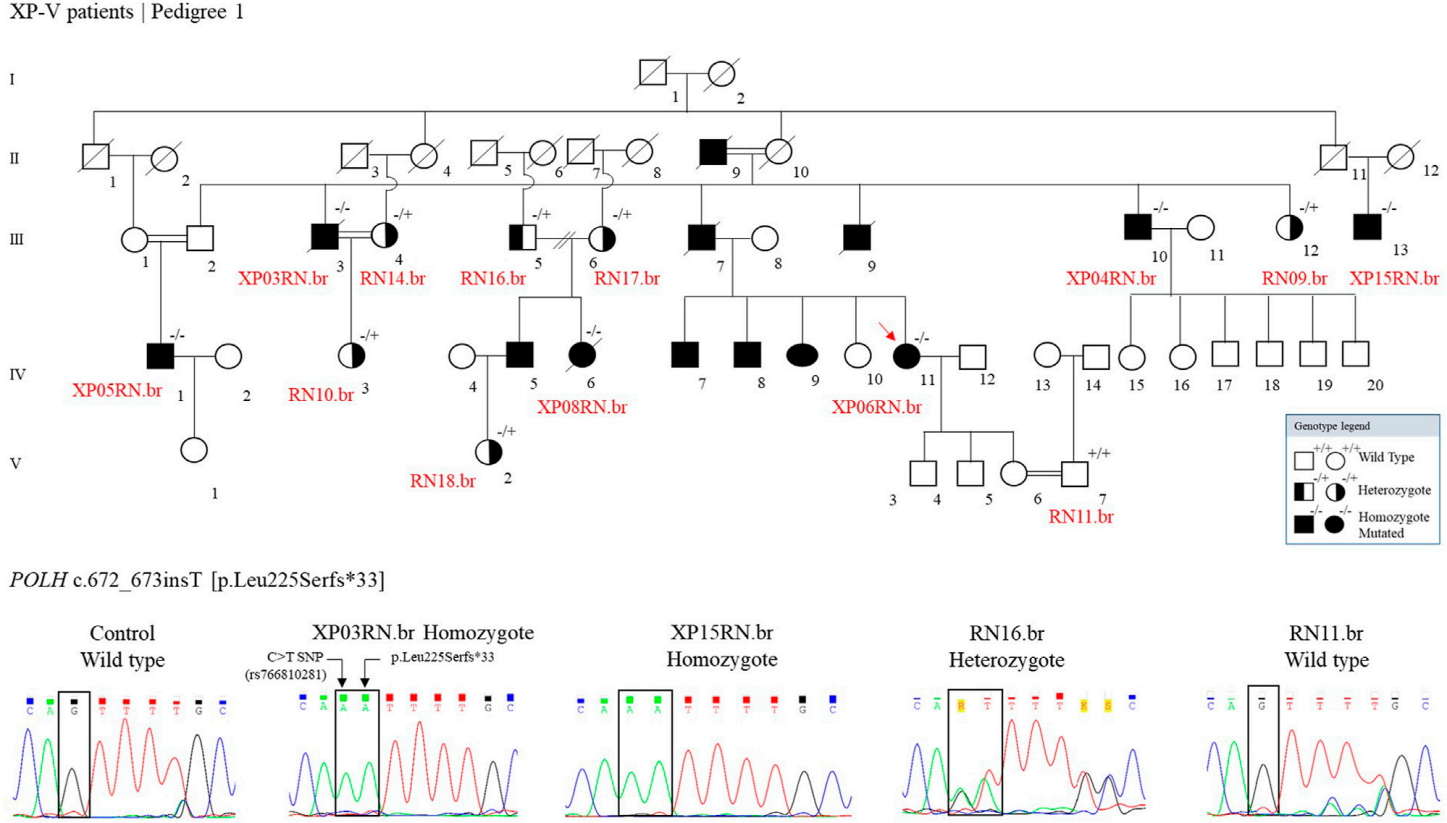
The genetic segregation of the p.Leu225Serfs*33 mutation was confirmed in the families from the community. Genealogy and genotype from the XP-V family at Pedigree 1. Electropherogram picture of the Sanger reaction at the *POLH* mutation site (reverse strand). The p.Leu225Serfs*33 comes alongside a C > T polymorphism (rs766810281) that leads to a synonymous exchange (p.Leu225Leu) and is presented in the Latin American and European populations and is probably segregating with the mutation.

We genotyped 13 individuals from pedigree 1: 1 wild type, 6 heterozygotes, and 6 homozygotes mutated (Figure 3). Other XP patients are indicated in the figure, based on information from the family, with no genetic analysis for the mutation.

The Genome Aggregation database (gnomAD, https://gnomad.broadinstitute.org/) reported the p.Leu225Serfs*33 (rs772778835) variant in heterozygous state in Latin American (3 out of 34,592 alleles) and European (1 out of 113,736 alleles) populations with a total allele frequency of 0.000008 and 0.000016, respectively. Although the mutation was identified in a low frequency in heterozygosity in Latin American, it was not found in Brazilian databases of germline exonic variants (ABraOM/https://abraom.ib.usp.br/; SELAdb http://intranet.fm.usp.br/sela).

**FIGURE 4:**
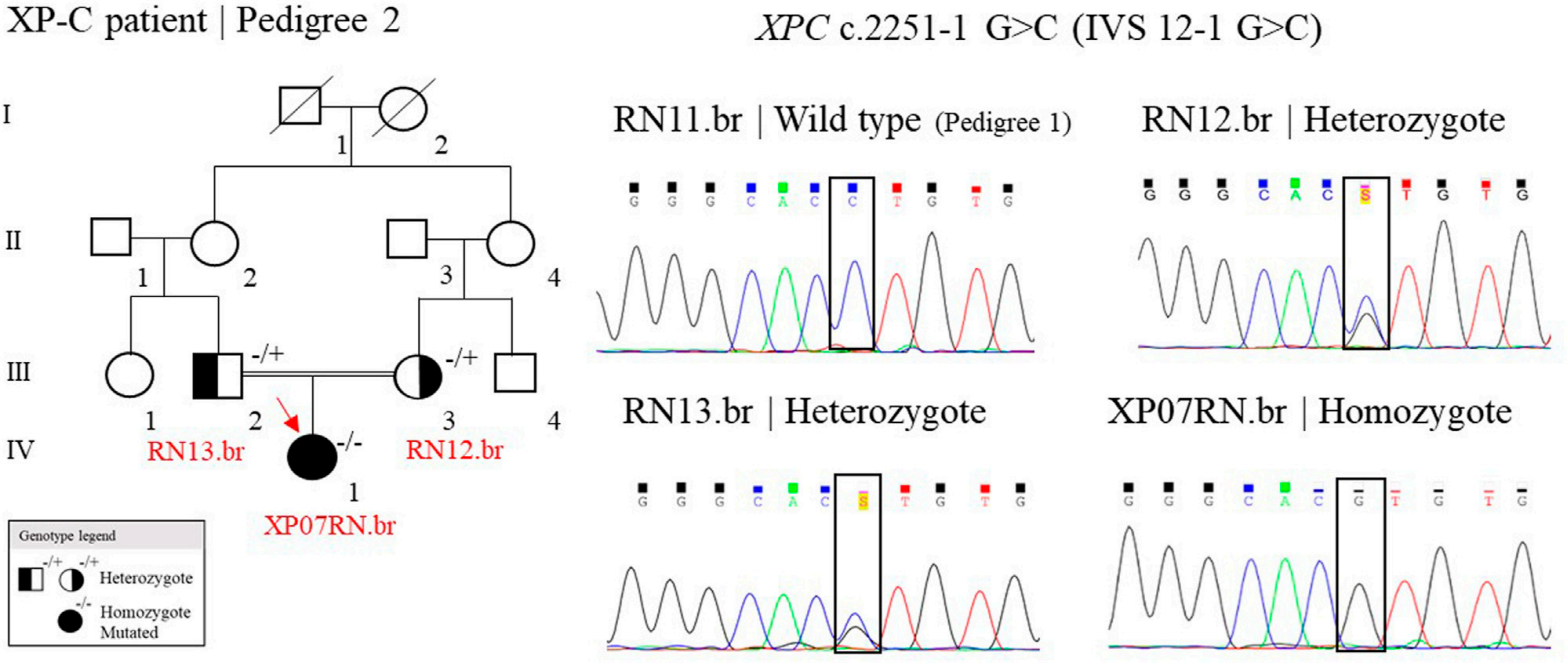
Genealogy and genotype from the XP-C family at Pedigree 2 and the electropherogram picture of the Sanger reaction at the XPC mutation site. Samples genotyped by Sanger have their ID. The red arrow indicates the patient sequenced by NGS. (+/+) represent wild type, (−/+) heterozygote, and (−/−) homozygote mutated.

One patient, out of seven, was homozygote for the splicing site mutation, c.2251-1 G>C, in the *XPC* gene (patient XP07RN.br, Figure 4)*.* This mutation was first described in individuals from the western Indian Ocean in Comoros, IVS 12–1 G>C, the “Comoros mutation” (Cartault et al., 2011), and is also present in the ClinVar database (ClinVar ID 190213). A further ancestry study indicated that these patients were derived from the Bantu group and had a founder effect on the Comoros island about 800 years ago (Sarasin et al., 2019a). At gnomAD and TOPMed databases, this splicing site mutation (rs754673606) was observed in heterozygosity with an allele frequency of 0.00002 and 0.00011, respectively, observed in the African/African-American and Latino/Admixed American population. This mutation has a high incidence in XP patients in Brazil: 15 cases out of 32 (Santiago et al., 2020). The Bantu African people, mainly located on the Atlantic coast of Africa (Congo and Angola) and on the Indian Ocean coast (Mozambique), were one of the major groups (90%) brought to Brazil during the 500 years of the slave trade (Lemos Cardoso and Farias Guerreiro, 2006). Thus, probably, this *XPC* mutation came with these slaves. Besides Comoros and Brazil, the mutation was also reported in Europe (in a patient with a Middle Eastern origin), Mozambique, and Pakistan (Fassihi et al., 2016; Ijaz et al., 2019; Kgokolo et al., 2019). The information on the ancestry of patients from Montanhas is almost null. When interrogated about the origin of their grandparents and great-grandparents, the patients reported that their families had always been from the region, and we were not able to determine the ethnic origins of these families. This story has probably been lost in the memory of the community.

This is the first report of an XP genetic cluster with two different mutated genes. The approach of NGS to carry out molecular diagnosis streamlined precision medicine care. After 50 years of molecular diagnosis and clinical findings on XP, we now know that the XP-C complementation group (NER-deficient) is particularly more prone to develop internal tumors than the other XP groups (Sarasin et al., 2019b). Cells from XP-V patients are NER-proficient, which would explain their milder phenotype, and the tumorigenesis is due to a defective mechanism of TLS during DNA replication. The difference in the molecular mechanism is directly linked to the clinical phenotype and prognosis of the patient. Establishing the culture of primary fibroblasts for functional assays, mainly from the patients with the novel insertion at *POLH*, could bring a better understanding of the molecular mechanisms involved with the clinic phenotype of these patients.

In Montanhas, none of the patients received educational materials to learn how to perform the appropriate photoprotection. Without financial conditions and government support, none of them protects themselves from sun exposure adequately. Even in this extreme condition, where the XP patients have lived under a high amount of sunlight exposure, only one XP-V (out of six) developed melanoma at 73 years of age, which is different from the XP-C patient, who was diagnosed with a melanoma at 7 years of age, indicating that the variation in clinical phenotypes observed in the XP patients might be due to the different mutated genes involved in their prognosis.

## Conclusion

In this work, we reported a small city in the Northeast region of Brazil (Montanhas, RN), with patients mutated at the *XPC* or *POLH* gene. The prognosis of the disease varies according to the mutated gene, the affected repair pathway, and the level of individual photoprotection. The XP-C patient had the most severe symptoms, which appeared before the first year of life, and a melanoma onset at 7 years old. For the XP-V patients, the mean age for the first symptoms was 16 years, and one developed melanoma at 73 years. The patients report minimal photoprotection using sporadic sunscreen when they have it. The mother of the XP-C is the most informed and worried about protection and prevention, mainly due to the high photosensitivity of the child. The difficulty in accessing medical care at the locality exacerbates the worst prognosis of a disease such as XP, especially the XP-C group, with an urgent need for continuous care and tumor excision. It should be noted that the low latitude of the location, near the equator line (approximately 445 miles), implicates high levels of DNA damage by sunlight (Schuch et al., 2012). There is an imperative demand on investing in prevention, providing them physical photoprotection and educational support, more importantly, facilitating access to medical care and prioritizing urgency for these patients.

## Data Availability

The mutations were reported at ClinVar, accession number VCV001119983.1 (NM_006502.3(POLH):c.672_673insT (p.Leu225fs), and SCV001652707.1 (NM_004628.5(XPC):c.2251-1G>C).
