# Supplementary material for: XPC and POLH/XPV Genes Mutated in a Genetic Cluster of Xeroderma Pigmentosum Patients in Northeast Brazil
Source: Front Genet. 2022 Jan 17;12:784963. doi: 10.3389/fgene.2021.784963 (PMC8801741; doi:10.3389/fgene.2021.784963)
Supplement: Supplementary file 1 [file DataSheet1.PDF]

Supplementary Material

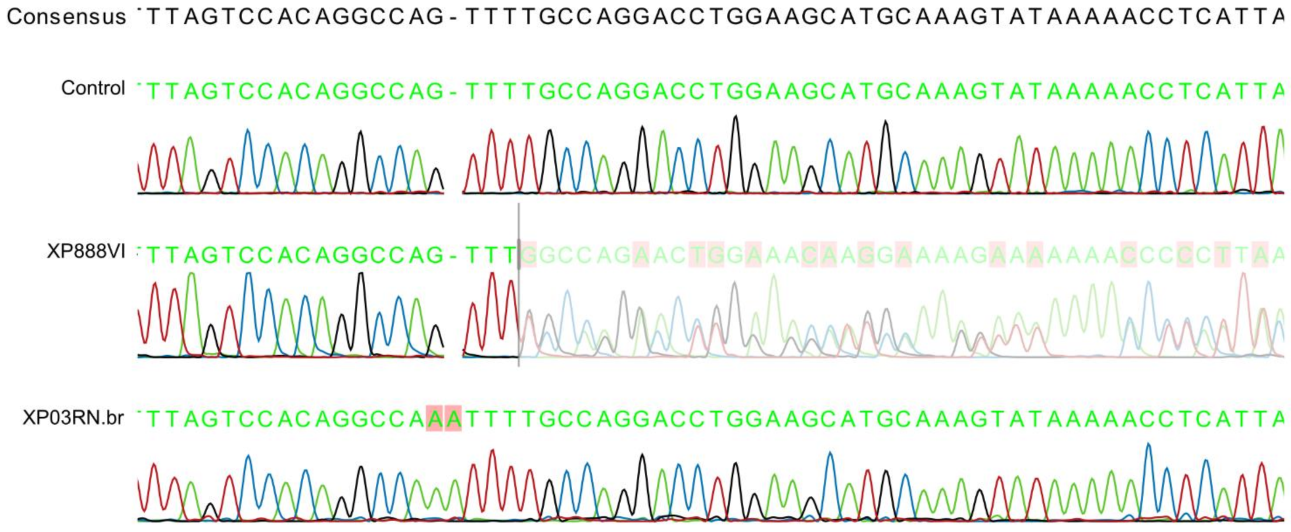

Supplementary Figure 1. A deletion at *POLH*, c.672delA [p.Lys224Trpfs229], close to the insertion that we identified c.672_673insT [p.Leu225Serfs*33], was reported in patients originated from North Africa. Electropherogram picture from the Sanger sequencing at the *POLH* mutation site (reverse strand) comparing the two samples XP888VI (heterozygous) from Algeria, and XP03RN.br (homozygous) from Brazil showed that indeed it refers to different alterations.
